# Supplementary material for: Consistent patterns of distractor effects during decision making
Source: eLife. 2020 Jul 6;9:e53850. doi: 10.7554/eLife.53850 (PMC7371422; doi:10.7554/eLife.53850)
Supplement: Supplementary file 1. — (A) Regression coefficients estimated using the analysis procedures suggested by Gluth et al., 2018. (B) Simulation 1: testing the probabilities of Type I and II errors when distractor effects were assumed to be absent in the simulated choice data. (C) Simulation 2: an alternative approach for testing the probabilities of Type I and II errors when distractor effects were assumed to be absent in the simulated choice data. (D) Simulation 3: testing the probabilities of Type I and II errors when distractor effects were assumed to be present in the simulated choice data. [file elife-53850-supp1.docx]

**Consistent patterns of distractor effects during decision making.**

**Bolton K. H. Chau, Chun-Kit Law, Alizée Lopez-Persem, Miriam C. Klein-Flügge, Matthew F. S. Rushworth**

**Supplementary File 1.**

| **Supplementary file 1A. Regression coefficients estimated using the analysis procedures suggested by Gluth et al. (2018).** | | | | | | |
| --- | --- | --- | --- | --- | --- | --- |
| Regressors | Exp 1  fMRI2014 | Exp 2  Gluth4 | Exp 4  Gluth3 | Exp 5  Gluth2  (HP) | Exp 6  Gluth1 | Exp 5  Gluth2  (LP) |
| Intercept | 1.104*** | 1.131*** | 1.081*** | 1.075*** | 0.946*** | 1.638*** |
| HV-LV | 0.574*** | 0.555*** | 0.450*** | 0.492*** | 0.430*** | 0.757*** |
| HV+LV | -0.175** | -0.154*** | -0.171** | -0.172** | -0.098* | -0.210*** |
| D-HV^1^ | -0.058 | 0.005 | -0.011 | -0.026 | -0.030 | -0.067 |
| (HV-LV)(D-HV)^2^ | -0.134** | -0.038# | -0.079** | 0.046 | -0.047# | -0.097* |
| D present^3^ | -0.119# | -0.208*** | -0.141* | -0.168* | -0.161* | 0.011 |
|  |  |  |  |  |  |  |
| 1. The term used by Gluth et al. (2018) is HV-D  2. The term used by Gluth et al. (2018) is (HV-LV)(HV-D)  3. A binary term indicating whether a distractor was present or not  Note that it is not possible to run the same analysis for the data of Exp3 and 7 as they lack two-option trials.  HP=high-time pressure experiment; LP=low-time pressure experiment  # *p*<0.1, * *p*<0.05, ** *p*<0.01, *** *p*<0.001 | | | | | | |

| **Supplementary file 1B. Simulation 1: testing the probabilities of Type I and II errors when distractor effects were assumed to be absent in the simulated choice data.** | | | | | | | |
| --- | --- | --- | --- | --- | --- | --- | --- |
| GLM | Regressors | Effect assumed | *β* | *t* | *p* | P (Type I error) | P (Type II error) |
| 1a | Intercept | Yes | 1.037 | 19.865 | <10^-37^ | -- | 0% |
|  | HV-LV | Yes | 0.617 | 12.463 | <10^-21^ | -- | 0% |
|  | D-HV | No | 0.009 | 0.383 | 0.702 | 5.9% | -- |
|  | (HV-LV)(D-HV) | No | 0.010 | 0.435 | 0.665 | 5.2% | -- |
|  |  |  |  |  |  |  |  |
| 1b | Intercept | Yes | 1.006 | 21.666 | <10^-39^ | -- | 0% |
|  | HV-LV | Yes | 0.573 | 14.717 | <10^-26^ | -- | 0% |
|  | HV+LV | Yes | -0.027 | -0.492 | 0.623 | -- | 47.4% |
|  | D-HV | No | 0.021 | 0.580 | 0.563 | 4.8% | -- |
|  | (HV-LV)(D-HV) | No | -0.006 | -0.264 | 0.792 | 5.1% | -- |
|  |  |  |  |  |  |  |  |
| 5 | Intercept | Yes | 1.139 | 0.572 | <10^-37^ | -- | 0% |
|  | HV-LV | Yes | 0.677 | 0.824 | <10^-13^ | -- | 0% |
|  | HV+LV | Yes | -0.689 | 0.741 | <10^-16^ | -- | 0% |
|  | D | No | -0.011 | 0.277 | 0.686 | 4.2% | -- |
|  | (HV-LV)D | No | 0.062 | 0.476 | 0.184 | 7.1% | -- |
|  | (HV+LV)D | No | 0.045 | 0.506 | 0.368 | 8.6% | -- |
|  | (HV-LV)(HV+LV) | Yes | -0.396 | 0.816 | <10^-5^ | -- | 0% |
|  | (HV-LV)(HV+LV)D | No | 0.097 | 0.505 | 0.051 | 6.0% | -- |
|  |  |  |  |  |  |  |  |
|  |  |  |  |  |  |  |  |

| **Supplementary file 1C. Simulation 2: an alternative approach for testing the probabilities of Type I and II errors when distractor effects were assumed to be absent in the simulated choice data.** | | | | | | | |
| --- | --- | --- | --- | --- | --- | --- | --- |
| GLM | Regressors | Effect assumed# | *β* | *t* | *p* | P (Type I error) | P (Type II error) |
| 1a | Intercept | Yes | 1.005 | 14.541 | <10^-16^ | -- | 0% |
|  | HV-LV | Yes | 0.420 | 8.150 | <10^-9^ | -- | 0% |
|  | D-HV | No | 0.021 | 0.776 | 0.442 | 4.9% | -- |
|  | (HV-LV)(D-HV) | No | -0.005 | -0.145 | 0.885 | 6.2% | -- |
|  |  |  |  |  |  |  |  |
| 1b | Intercept | Yes | 1.039 | 12.713 | <10^-14^ | -- | 0% |
|  | HV-LV | Yes | 0.464 | 9.235 | <10^-10^ | -- | 0% |
|  | HV+LV | Yes | -0.308 | -4.429 | <10^-4^ | -- | 1.0% |
|  | D-HV | No | -0.070 | -1.523 | 0.136 | 6.3% | -- |
|  | (HV-LV)(D-HV) | No | 0.002 | 1.674 | 0.952 | 5.6% | -- |
|  |  |  |  |  |  |  |  |
| 5 | Intercept | Yes | 1.079 | 10.162 | <10^-11^ | -- | 0% |
|  | HV-LV | Yes | 0.493 | 4.855 | <10^-4^ | -- | 0% |
|  | HV+LV | Yes | -0.246 | -2.150 | 0.038 | -- | 0% |
|  | D | No | -0.009 | -0.114 | 0.910 | 6.3% | -- |
|  | (HV-LV)D | No | 0.063 | 0.675 | 0.504 | 5.5% | -- |
|  | (HV+LV)D | No | -0.060 | -0.677 | 0.502 | 8.5% | -- |
|  | (HV-LV)(HV+LV) | Yes | -0.057 | -0.571 | 0.571 | -- | 0% |
|  | (HV-LV)(HV+LV)D | No | 0.055 | 0.694 | 0.492 | 6.2% | -- |
|  |  |  |  |  |  |  |  |
| # A subset of terms is assumed to have an effect in the simulated choice data. In this analysis the effect sizes of these terms with assumed effects are estimated from the empirical data using GLMs that include these terms only. By contrast in Supplementary Table 2, the full GLMs are applied to estimate the effect sizes. | | | | | | | |

| **Supplementary file 1D. Simulation 3: testing the probabilities of Type I and II errors when distractor effects were assumed to be present in the simulated choice data.** | | | | | | | |
| --- | --- | --- | --- | --- | --- | --- | --- |
| GLM | Regressors | Effect assumed | *β* | *t* | *p* | P (Type I error) | P (Type II error) |
| 1a | Intercept | Yes | 0.926 | 29.120 | <10^-74^ | -- | 0% |
|  | HV-LV | Yes | 0.584 | 19.511 | <10^-48^ | -- | 0% |
|  | D-HV | Yes | 0.101 | 4.450 | <10^-4^ | -- | 0.1% |
|  | (HV-LV)(D-HV) | Yes | -0.109 | -4.435 | <10^-4^ | -- | 4.7% |
|  |  |  |  |  |  |  |  |
| 1b | Intercept | Yes | 0.941 | 28.494 | <10^-72^ | -- | 0% |
|  | HV-LV | Yes | 0.604 | 21.646 | <10^-54^ | -- | 0% |
|  | HV+LV | Yes | -0.118 | -3.949 | <10^-3^ | -- | 29.8% |
|  | D-HV | Yes | 0.017 | 0.583 | 0.561 | -- | 26.3% |
|  | (HV-LV)(D-HV) | Yes | -0.100 | -4.286 | <10^-4^ | -- | 0.6% |
|  |  |  |  |  |  |  |  |
| 5 | Intercept | Yes | 1.049 | 26.364 | <10^-67^ | -- | 0% |
|  | HV-LV | Yes | 0.718 | 14.444 | <10^-32^ | -- | 0% |
|  | HV+LV | Yes | -0.476 | -9.654 | <10^-17^ | -- | 0% |
|  | D^#^ | No | 0.019 | 0.761 | 0.448 | 2.4% | -- |
|  | (HV-LV)D | Yes | -0.170 | -3.894 | <10^-3^ | -- | 17.8% |
|  | (HV+LV)D | Yes | 0.481 | 10.259 | <10^-19^ | -- | 0% |
|  | (HV-LV)(HV+LV) | Yes | -0.286 | -6.120 | <10^-8^ | -- | 0% |
|  | (HV-LV)(HV+LV)D | Yes | 0.403 | 7.612 | <10^-12^ | -- | 0% |
|  |  |  |  |  |  |  |  |
| # Effect not assumed because the effect was absent in the empirical data (Appendix 3-figure 2a). | | | | | | | |
