# Supplementary material for: Consistent patterns of distractor effects during decision making
Source: eLife. 2020 Jul 6;9:e53850. doi: 10.7554/eLife.53850 (PMC7371422; doi:10.7554/eLife.53850)
Supplement: Transparent reporting form [file elife-53850-transrepform.docx]

***eLife’s* transparent reporting form**

We encourage authors to provide detailed information *within their submission* to facilitate the interpretation and replication of experiments. Authors can upload supporting documentation to indicate the use of appropriate reporting guidelines for health-related research (see [EQUATOR Network](http://www.equator-network.org/%20)), life science research (see the [BioSharing Information Resource](https://biosharing.org/)), or the [ARRIVE guidelines](http://www.plosbiology.org/article/info:doi/10.1371/journal.pbio.1000412) for reporting work involving animal research. Where applicable, authors should refer to any relevant reporting standards documents in this form.

If you have any questions, please consult our Journal Policies and/or contact us: [editorial@elifesciences.org](mailto:editorial@elifesciences.org).

**Sample-size estimation**

- You should state whether an appropriate sample size was computed when the study was being designed
- You should state the statistical method of sample size computation and any required assumptions
- If no explicit power analysis was used, you should describe how you decided what sample (replicate) size (number) to use

Please outline where this information can be found within the submission (e.g., sections or figure legends), or explain why this information doesn’t apply to your submission:

This study did not involve an *a priori* sample size estimation. Part of the reason was because 5 out of 8 experiments involved data obtained from previously published datasets. However, we still included in our manuscript a power analysis to confirm that each experiment involved a sufficiently large sample size. This is included in the section *Methods, Human Participants* (P.20):

*We conducted a posteriori power analysis to confirm that the sample sizes of the experiments were adequate. All analyses were conducted using criteria of power=80% and alpha=0.05 two tailed. Experiments 2-6 were replicates of Experiment 1 in which they all tested whether there was a difficulty-dependent distractor effect via the (HV-LV)(D-HV) term in GLM1a. In Experiment 1, the effect size in Cohen’s d for the (HV-LV)(D-HV) effect was d=0.790. The required sample size was calculated at 13 participants and Experiments 1-6 all involved larger samples. Experiment 7 was conducted simultaneously with Experiment 3 in which it tested the salience-based effect of the distractor via the |D| term in GLM6a. The effect size in Cohen’s d for the |D| effect was d= 0.514. The required sample size was calculated at 30 participants and the sample of Experiment 7 exceeded this size.* *Experiment 8 examined the impact of attentional capture on decision making. One key analysis was to examine whether more gaze shifts from D to HV were related to greater accuracies (GLM9), in which the effect size in Cohen’s d was 0.607. The required sample size was calculated at 22 participants.* *The sample size of Experiment 8, after excluding four participants using the inclusion criterion of data validity >85% (see Eye tracking experiment procedures), was one participant less than this number estimated in a posteriori power analysis. However, when we relaxed the inclusion criterion to >70% data validity, a total of 22 participants were included and the results remained similar (Figure 8 and Figure 8-Figure Supplement 1).*

**Replicates**

- You should report how often each experiment was performed
- You should include a definition of biological versus technical replication
- The data obtained should be provided and sufficient information should be provided to indicate the number of independent biological and/or technical replicates
- If you encountered any outliers, you should describe how these were handled
- Criteria for exclusion/inclusion of data should be clearly stated
- High-throughput sequence data should be uploaded before submission, with a private link for reviewers provided (these are available from both GEO and ArrayExpress)

Please outline where this information can be found within the submission (e.g., sections or figure legends), or explain why this information doesn’t apply to your submission:

The study mainly included a decision making task that was replicated or adapted across 8 experiments. In the section *Methods, Summary of approach*, it summarizes how the dataset of each experiment was collected and it also describes the difference between experiments.

An outlier was identified when GLM5 was applied to analyze data from Experiment 8; the data point was larger than the mean by 4.1 times the standard deviation (Page 15, Lines 681-688).

The quality of the eye movement data was estimated moment-by-moment using an in-built algorithm of the Tobii TX300 eye tracker. The inclusion/exclusion criteria of the eye movement data were described in the section *Methods, Eye tracking eye* (Page 22, Lines 1039-1054).

**Statistical reporting**

- Statistical analysis methods should be described and justified
- Raw data should be presented in figures whenever informative to do so (typically when N per group is less than 10)
- For each experiment, you should identify the statistical tests used, exact values of N, definitions of center, methods of multiple test correction, and dispersion and precision measures (e.g., mean, median, SD, SEM, confidence intervals; and, for the major substantive results, a measure of effect size (e.g., Pearson's r, Cohen's d)
- Report exact p-values wherever possible alongside the summary statistics and 95% confidence intervals. These should be reported for all key questions and not only when the p-value is less than 0.05.

Please outline where this information can be found within the submission (e.g., sections or figure legends), or explain why this information doesn’t apply to your submission:

Explanations of the statistical analysis methods are provided in *Methods, Analysis Procedure* (Page 22) and also repeated in various sections of *Results*.

The sample sizes of all experiments were all larger than 20. Error bars indicating standard errors were presented in all bar charts.

The statistical test used and the sample size of each test (in form of degree-of-freedom) were described in both the *Results* and *Methods* sections. The mean and standard error of the key results can be found in the figures in form of bar charts.

The exact p values of each test were reported to 3 decimal places in the *Results* section. Exceptions are the two analyzes there were repeated across three experiments (Line 464) and six experiments (Line 475) respectively. The largest p value, across experiments, of each analysis was indicated.

(For large datasets, or papers with a very large number of statistical tests, you may upload a single table file with tests, Ns, etc., with reference to sections in the manuscript.)

**Group allocation**

- Indicate how samples were allocated into experimental groups (in the case of clinical studies, please specify allocation to treatment method); if randomization was used, please also state if restricted randomization was applied
- Indicate if masking was used during group allocation, data collection and/or data analysis

Please outline where this information can be found within the submission (e.g., sections or figure legends), or explain why this information doesn’t apply to your submission:

Not applicable, since all experiments involved a within-subject design with no group allocation.

**Additional data files (“source data”)**

- We encourage you to upload relevant additional data files, such as numerical data that are represented as a graph in a figure, or as a summary table
- Where provided, these should be in the most useful format, and they can be uploaded as “Source data” files linked to a main figure or table
- Include model definition files including the full list of parameters used
- Include code used for data analysis (e.g., R, MatLab)
- Avoid stating that data files are “available upon request”

Please indicate the figures or tables for which source data files have been provided:

The codes for running the mutual inhibition model, divisive normalization model, dual route model and null model can be found at:

<https://doi.org/10.5061/dryad.k6djh9w3c>

Behavioural data of Experiments 1, 3 and 7 can be found at:

<https://datadryad.org/stash/dataset/doi:10.5061/dryad.040h9t7>

Behavioural data of Experiments 2, 4-6 can be found from a link provided by Gluth and colleagues (2018) at:

<https://osf.io/8r4fh/>

Behavioural and eye tracking data of Experiment 8 can be found at:

<https://doi.org/10.5061/dryad.k6djh9w3c>
